# Supplementary material for: Korean Red Ginseng-Induced SIRT3 Promotes the Tom22–HIF-1α Circuit in Normoxic Astrocytes
Source: Cells. 2023 May 30;12(11):1512. doi: 10.3390/cells12111512 (PMC10252242; doi:10.3390/cells12111512)
Supplement: Supplementary file 1 [file cells-12-01512-s001.zip › cells-2385024-supplementary.pdf]

Supplementary data

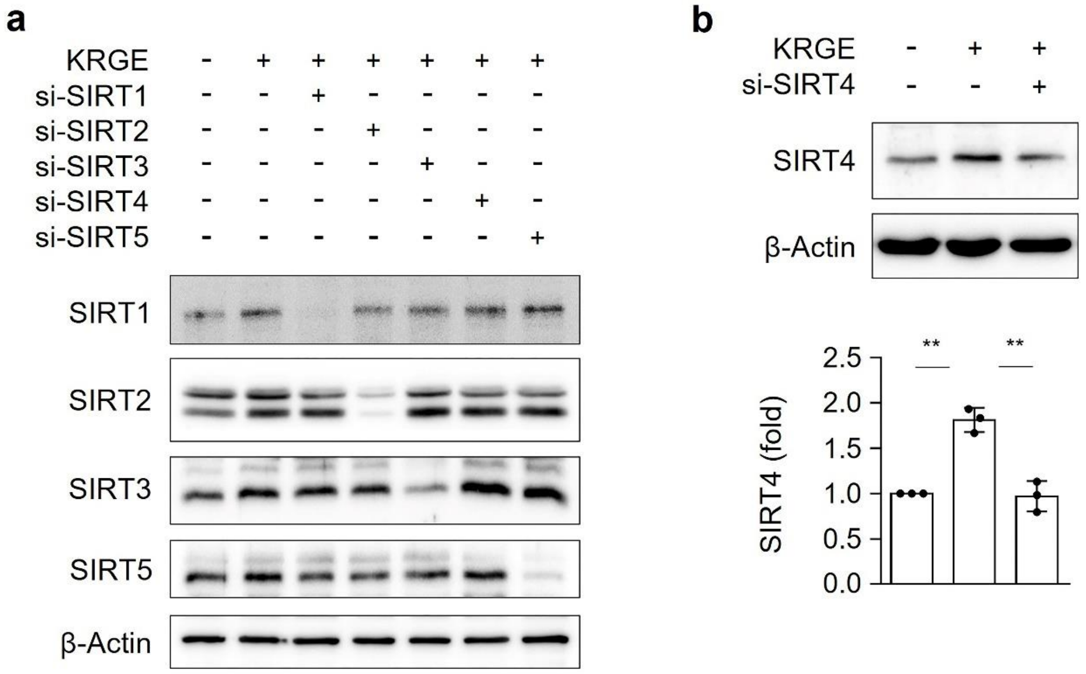

Supplementary Figure S1. Transfection efficiency was shown when astrocyte cells were transfected with indicated si-RNA. (a-b) Astrocytes were transfected with control or indicated si-RNA and subjected to 0.5 mg/ml KRGE for 24 h. Target protein levels in cell lysate were detected via western blotting (n = 3-6). $^{**}P < 0.01$.

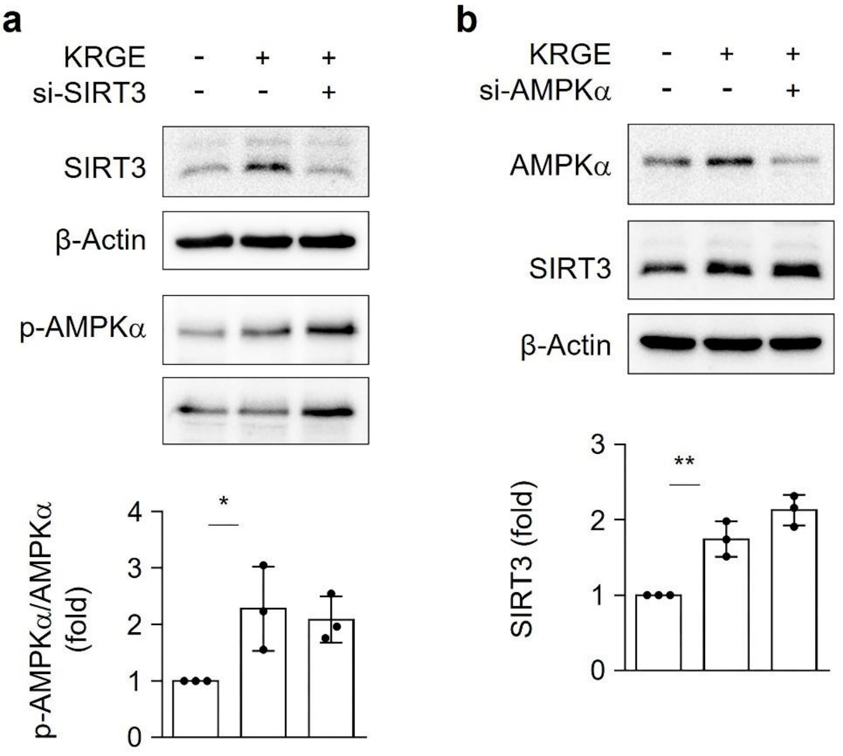

Supplementary Figure S2. KRGE-induced SIRT3 did not regulate AMPKα activation and *vice versa* in astrocytes cells. Astrocytes were transfected with control or indicated si-RNA and subjected to 0.5 mg/ml KRGE for 24 h. (a)

p-AMPKα, AMPKα ($n = 3$) and (b) SIRT3 ($n = 3$) were detected via western blotting. Relative p-AMPKα/AMPKα ratio and SIRT3 levels were quantified. $^{*}P < 0.05$: $^{**}P < 0.01$.
